# Dental practice satisfaction with preferred provider organizations

**DOI:** 10.1186/1472-6963-7-184

**Published:** 2007-11-15

**Authors:** Robert H Aseltine, Susan Reisine, Elizabeth A Schilling, James Kennedy

**Affiliations:** 1 University of Connecticut School of Dental Medicine, Department of Oral Health and Diagnostic Sciences, Division of Behavioral Sciences and Community Health, MC 3910, 263 Farmington Avenue, Farmington, CT 06030-3910, USA; 2 Institute for Public Health Research, University of Connecticut, MC 7160, 99 Ash Street, East Hartford, CT 06108, USA; 3 University of Connecticut School of Dental Medicine, Department of Oral Health and Diagnostic Sciences, Division of Periodontology, MC 3910, 263 Farmington Avenue, Farmington, CT 06030-3910, USA

## Abstract

**Background:**

Despite their increasing share of the dental insurance market, little is known about dental practices' satisfaction with preferred provider organizations (PPOs). This analysis examined practice satisfaction with dental PPOs and the extent to which satisfaction was a function of communications from the plan, claims handling and compensation.

**Methods:**

Data were collected through telephone surveys with dental practices affiliated with MetLife between January 2002 and December 2004. Each respondent was asked a series of questions related to their satisfaction with a systematically selected PPO with which they were affiliated. Six different PPO plans had sufficient observations to allow for comparative analysis (total n = 4582). Multiple imputation procedures were used to adjust for item non-response.

**Results:**

While the average level of overall satisfaction with the target plan fell between "very satisfied" and "satisfied," regression models revealed substantial differences in overall satisfaction across the 6 PPOs (p < .05). Statistically significant differences between plans in overall satisfaction were largely explained by differences in the perceived adequacy of compensation. However, differences in overall satisfaction involving two of the PPOs were also driven by satisfaction with claims handling.

**Conclusion:**

Results demonstrate the importance of compensation to dental practice satisfaction with PPOs. However, these results also highlight the critical role of service-related factors in differentiating plans and suggest that there are important non-monetary dimensions of PPO performance that can be used to recruit and retain practices.

## Background

Approximately 159 million Americans have some form of dental insurance covering about 54% of the United States population and 62% of patients in private dental practices in the United States [1,2]. Unlike insurance for medical care which is based on principles of risk, dental insurance typically consists of payment plans that help individuals by paying for a portion of the cost of their dental care. However, according to a recent report by the National Association of Dental Plans,[1] the composition of the dental insurance market has changed dramatically in the past decade. Fee-for-service indemnity structures dominated dental reimbursement programs in the early 1990s, with 70% of the population being covered by such plans in 1994. Today, only about a quarter of the population is covered by indemnity plans. Managed care in dentistry, particularly preferred provider organizations (PPOs), has seen rapid growth in recent years [1,3,4]. The number of people covered by PPOs increased from 11.4 million in 1994 to 79.7 million in 2004, which constitutes a five-fold increase in market share over the past 10 years.

Many of the same constraints physicians experienced in the rapid growth of managed care programs in medicine have confronted dentists as insurance coverage and managed care programs expanded in dentistry. As dental insurance coverage increased in the 1970s and 1980s, dentists, like physicians, expressed concerns about threats to professional autonomy and limits on clinical judgment in the management of patient care [5-7]. More recently, studies of physician satisfaction with managed care have assessed factors other than professional autonomy as major influences on physician satisfaction with both career outcomes and managed care programs. These other factors included reimbursement rates, referrals, incentive payments and time pressure [8-11]. Most studies reported that a majority of physicians were dissatisfied with managed care and that dissatisfaction was increasing. Physicians were most dissatisfied with control of referrals, gate-keeping and denial of care. Additionally, physicians were unhappy about financial aspects of managed care, including declining income and reimbursement levels and the structure of financial incentives.

Relatively few studies of dentists' and/or their office staffs' perceptions of dental insurance generally, and managed care specifically, exist. In 1995, the American Dental Association undertook a major survey of its members to assess the demographic characteristics of dentists participating in managed care programs [12], dentists' views of managed care [13], and differences between dentists who participate in capitation and PPO programs [14]. Thirty-one percent (31%) of respondents reported participating in at least one managed care contract. Participation was highest in the Pacific region (40.1%) and lowest in New England (24%). Males, those in solo practice and those in practice longer were less likely to participate in managed care compared to females, non-solo practice and those in practice less than ten years. Those who did not participate in managed care tended to have higher incomes, but this could be due to other factors such as being in practice longer. Participation in managed care was also associated with practice patterns. Dentists participating in managed care worked slightly more hours, had more patients per week, fewer visits per patient and shorter appointments compared to those with no managed care contracts.

The analysis of dentists' views of managed care indicated that dentists, whether or not they participated in managed care plans, believed that managed care "does not reimburse dentists adequately" and that managed care "is not in the best interest of patients." However, dentists participating in managed care were more likely to agree with statements that "managed care will fill available chair time, does not interfere with the doctor-patient relationship and that managed care is good for dentists just starting out." Analysis of the data on comparisons of capitation and PPO plans showed that participants in both type of plans reported that the main incentive for joining was expanding their patient base and the main deterrent was unattractive fee schedules. Only 7% cited lack of control over patient treatment options as a reason for not joining a managed care plan. Results from the three publications based on the ADA survey suggested that financial incentives and reimbursement were prime issues for dentists in their decision to join a PPO or capitation plan. This was particularly true for younger dentists starting out in practice. Surprisingly, issues related to professional autonomy did not seem to be as important as earlier studies might suggest, especially when compared to results of studies on career satisfaction among primary care physicians.

Public demand for dental insurance is increasing and managed care structures seem to be the most feasible approach to expanding access to dental benefits. Witt and colleagues [3] reported that dental benefits were the most desired benefit among employees after medical benefits and that many employers that do not currently offer dental benefits planned to offer them in the next two years. Given the increasing demand for dental insurance and the attractiveness of PPO plans in terms of cost, provider choice and flexibility, more information is needed on factors associated with dental practices' satisfaction with managed care programs in order to encourage enrollment and maintain participation in these plans. To address this question, we analyzed data from a recent national survey of dental practices affiliated with the 6 largest dental PPOs in the US. Our analysis examined two interrelated questions. First, to what extent is overall satisfaction with dental PPOs a function of ratings of *communications *from the plan (e.g., clarity of claim payment guidelines; clarity of EOB statements), *claims handling *(e.g., claims review policy; timeliness of payments), and overall *compensation*? Second, to what extent can differences in overall levels of satisfaction with particular PPOs be explained by these 3 factors?

## Methods

This study consisted of a secondary analysis of survey data collected from dental practices affiliated with MetLife, which among its array of health and dental insurance plans includes a dental PPO. Telephone surveys were conducted on a monthly basis between January 2002 and December 2004 with representatives from practices submitting claims to MetLife in the previous 12 months. Practices were told that the surveys were being conducted "on behalf of the dental industry;" MetLife was never identified as the sponsor of the surveys. Data were collected by Zeldis Research Associates. Respondents included dentists or office managers, with the latter included as targets of the survey due to their firsthand knowledge of factors such as the timeliness of payments and overall claim service. Although the 2002 to 2004 surveys did not code the identity of the respondent, responses from surveys conducted in the first 3 months of 2006 (which did capture this information) indicated that approximately 80% of respondents were office managers.

Quota sampling was used to allocate practices to dental PPOs with which they were affiliated. The first 100 dental practices participating in the survey were asked to rate their experience with MetLife; the next 50 practices were asked to enumerate the 3 PPOs to which they submitted the most claims and were then randomly assigned to rate one of the these plans. A total of 14 distinct PPO plans were enumerated by 5294 practices surveyed over the 3 year period. To ensure a sufficient number of cases for analysis, this study excluded all PPOs that did not obtain ratings from at least 75 practices, which eliminated 712 practices rating 8 different PPO plans. As a result our analysis was based on ratings of 6 different PPO plans obtained from 4582 practices. With the exception of the oversampling of practices affiliated with MetLife, the distribution of practices among plans was virtually identical to what would be expected based on market share data provided in each company's annual report and/or marketing materials.

Because this study involved secondary analysis of data that were collected anonymously and included no identifying information, it did not constitute human subjects research under U.S. Department of Health and Human Services regulations 45 CFR part 46.

### Measures

The dependent variable for this analysis was a single item measuring overall satisfaction with the targeted dental PPO plan. Respondents were asked "Overall, how satisfied are you with [company PPO];" responses ranged from "very satisfied" to "very dissatisfied" on a 4-point scale. Three scales measuring different aspects and features of the target plan that were considered components of overall satisfaction were also included in the analysis. Items in all scales were rated using the same 4-point response scale used to assess overall satisfaction ("very satisfied" to "very dissatisfied"). *Claims Service *consisted of responses to the following four items: "Thinking about the claims service you receive from [company] over the past 12 months how would you rate: (a) the overall handling of your claims, (b) the claims review policy, (c) the amount of time it takes to receive payment of claims, and (d) the amount paid was correct based on your understanding of the plan." *Communication *consisted of responses to the following three items: "Thinking about the communication you received from [company] over the past 12 months how would you rate: (a) the communication you receive concerning claim payment guidelines, (b) your patients' understanding of their benefits, and (c) the clarity of EOB (Explanation of Benefit) statements." *Compensation *was measured by responses to the following two items: (a) "How satisfied are you with the overall compensation for your participation in the [company] PPO? This includes fee schedule, periodic exams, and claim reviews," and (b) "How satisfied are you with the [company] PPO fee schedule?" For each of these scales the component items were summed and divided by the number of items comprising the scale.

Finally, demographic characteristics of the participating practices – the number of full time dentists, the number of full time hygienists, the number of years in business, and the number of managed care plans the practice was affiliated with – were obtained in the 2003 and 2004 surveys. This information, however, was not included in the 2002 survey.

### Missing Values

While the percentage of cases with missing values was very low for the 4 measures of satisfaction, the omission of the practice demographics questions from the 2002 survey resulted in a large amount of missing data. To address this problem, we performed multiple imputation [15], a simulation-based approach that generates multiple plausible values for each missing element in order to represent the inherent uncertainty in the missing data [16]. We used the Markov Chain Monte Carlo (MCMC) method in SAS 9.0 to produce 10 imputed data sets that were subsequently analyzed using regression and MIANALYZE procedures in SAS.

## Results

Diagnostic plots indicated that multiple imputation using MCMC converged well. Between .3 and 2.5% of items measuring overall satisfaction and its components (median of 1%) had missing values because the respondent had refused to answer the question or answered "don't know." Approximately 27% of the demographic characteristics had missing values, almost all of which was due to the omission of these questions in the 2002 survey.

The demographic characteristics of practices participating in the survey are presented in Table 1. Practices had on average 1.5 full time dentists and 1.0 full time hygienists; approximately 17 years in business, and were affiliated with 7.7 dental managed care plans. Table 2 presents descriptive statistics and bivariate correlations for the major variables included in this analysis. Cronbach's alpha coefficients for the three scales measuring components of overall satisfaction – claims service, communication, and compensation – were .85, .70, and .81, respectively. The average level of overall satisfaction with the PPO respondents were asked to rate (i.e., the target plan) was 3.2, which fell between the "very satisfied" and "satisfied" response categories for this measure. Moderate associations among the components of overall satisfaction with the target PPO were observed, with correlations ranging between .48 and .66. In addition each of these variables was moderately associated with overall satisfaction with the target PPO.

**Table 1:** Demographic characteristics of practices using multiply imputed data (N = 4582)

Variable	Mean	*SD*
# FT Dentists	1.5	1.1
# FT Hygienists	1.0	1.3
# Years in business	16.9	11.5
# Managed care plans^a^	7.7	6.9

^a^The number of managed care plans combines the number of PPOs and dental health maintenance organizations.

**Table 2:** Descriptive statistics and bivariate associations among study variables, by plan

			Plan															
	Total		A		B		C		D		E		F		Correlations			

	M	SD	M	SD	M	SD	M	SD	M	SD	M	SD	M	SD	1.	2.	3.	4.

1. Overall satisfaction	3.2	.7	3.0	.7	3.1	.7	3.1	.6	3.3	.7	3.2	.7	3.3	.6	---			
2. Claims service	3.1	.5	2.9	.5	3.1	.5	3.1	.5	3.2	.5	3.1	.5	3.2	.5	.53	---		
3. Communication	3.1	.5	2.9	.6	3.0	.6	3.1	.5	3.2	.5	3.1	.5	3.1	.5	.44	.66	---	
4. Compensation	3.0	.7	2.7	.6	2.8	.6	2.9	.6	3.1	.6	2.9	.7	3.0	.6	.58	.50	.48	---

Pearson correlation coefficients are presented, all of which are significant at the .05 level.

To examine differences among PPOs in overall levels of satisfaction, Table 3 (Model 1) presents combined results using the 10 imputed datasets from regression analyses in which satisfaction was regressed on a series of dummy variables for plan. Coefficients reflect contrasts with the weighted sample mean on overall satisfaction, with the number of cases within each plan adjusted by overall PPO market share. The intercept term was suppressed in this analysis to obtain mean contrasts for all 6 plans. Regression diagnostics indicated that the model provided a good fit to the data as indicated by predicted value, residual, and studentized residual plots. Only 6% of predicted values fell outside the range (1 – 4) of the dependent variable; all of the predicted values falling outside this range exceeded the maximum value for this variable (4), with none higher than 4.19. Results from this analysis indicated substantial variability in overall ratings. Ratings of satisfaction with Plans A, B, and C fell below the overall sample mean, with the first 2 of these contrasts achieving statistical significance, while ratings for D, E, and F were higher than the sample mean, with the contrasts for D and F achieving statistical significance. Finally, the R^2 ^coefficient for this model was .027, indicating that while there were significant differences between plans in overall satisfaction, neither the differences among plans nor the practice demographics included in this model explained much of the variability in overall satisfaction.

**Table 3:** Plan differences in overall satisfaction, controlling for ratings of claims service, communication, and compensation, using multiply imputed data

	**Model 1**		**Model 2**	
	B	SE	B	SE
Plan A (n = 466)	-.152*	.029	.022	.024
Plan B (n = 800)	-.103*	.024	-.073*	.019
Plan C (n = 428)	-.057	.030	-.065*	.024
Plan D (n = 1841)	.138*	.019	.015	.015
Plan E (n = 801)	.046	.024	.048*	.018
Plan F (n = 246)	.128*	.038	.052	.030
				
Claims Service	---	---	.373*	.022
Communication	---	---	.077*	.020
Compensation	---	---	.441*	.015
R2	.027		.427	

*p < .05

All models were estimated using 10 datasets containing imputed values for cases with missing data (total N = 4582 in each dataset). Coefficients are calculated as deviations from the grand mean of the sample. Market share adjusted Ns for plans are presented in parentheses to adjust to for oversampling.

To determine the extent to which claims service, communication, and compensation accounted for the differences in overall satisfaction among plans, these potential mediating variables were added to the equation presented in Model 1. Results presented in Model 2 of Table 3 indicate that all three of variables were significantly related to overall satisfaction in the expected direction (e.g., higher ratings of the target plans' claims service were associated with higher levels of overall satisfaction with the target plan). Standardized coefficients (not shown) indicate that the associations between compensation and overall satisfaction (B = .44) and claims service and overall satisfaction (B = .37) were particularly strong, while the association between communication and satisfaction, although statistically significant, was quite weak (B = .08). The R^2 ^coefficient in this model (.427) is substantially higher than in Model 1, indicating that over 40% of the variability in overall satisfaction was explained by compensation, communication and claims service.

Controlling for these potential mediators had a substantial impact on plan differences in overall satisfaction. The coefficients reflecting plan differences were, with two exceptions, substantially reduced: for instance, the difference between Plan D and the overall mean was reduced by 89% (i.e., [.138-.015]/.138) in Model 2 when the 3 potential mediators were controlled. Only 3 plans – B, C and E – continued to differ significantly from the sample mean when these variables were controlled; however, significance tests for the *change *in plan coefficients from Model 1 to Model 2 indicate that statistically significant changes were limited to Plans A and D.

These results suggest that differences in overall satisfaction with dental PPOs were largely explained by differences in participants' ratings of the claims service, quality of communication, and compensation provided by these plans. However, they did not identify the particular factor or factors that accounted for overall differences in satisfaction. To determine the relative importance of these three factors in explaining the differences in satisfaction among plans, we calculated the indirect effects of plan on satisfaction separately through each explanatory variable. The indirect effects were calculated by subtracting the direct effect of plan from the plan coefficients obtained in a series of models dropping each mediator variable [17,18]. Results from this analysis are presented in Table 4. Although the indirect effects presented in this table indicate that compensation tended to play a central role in differences in overall satisfaction among plans, claims service was also critical for 2 of the plans. For instance, the largest indirect effect for Plan A involved compensation (-.034), but this was followed closely by claims service (-.023). The negative coefficients for these indirect effects indicate that Plan A was given substantially lower ratings of overall satisfaction due to their lower compensation rates and poorer claims service. Similar results were observed for Plan F, although here the impact of claims service (.015) is approaches the impact of compensation (.019) in explaining why this particular plan was given better satisfaction ratings than the average plan. The only other 2 plans differing substantially from the sample mean in overall satisfaction – B and D – did so almost solely because of differences in compensation: for Plan B, lower levels of satisfaction were accounted for by lower reimbursement rates, while higher levels of compensation accounted for the better ratings garnered by Plan D.

**Table 4:** Decomposing differences among plans in overall satisfaction by compensation, communication, and claims service

	PLAN					
	A	B	C	D	E	F
Total Effect	-.152*	-.103*	-.057	.138*	.046	.128*
Direct Effect	.022	-.073*	-.065*	.015	.048*	.052
						
Indirect Effects Through:						
Compensation	-.034	-.028	-.001	.044	.001	.019
Communication	-.002	.001	.000	.003	.001	-.002
Claims Service	-.023	.006	.005	-.001	-.004	.015

* p < .05

^a ^The total effect is the plan coefficient from the reduced model presented in Table 3 (Model 1).

^b ^The direct effect is the plan coefficient from the full model presented in Table 3 (Model 2).

^c ^The indirect effects are the arithmetic differences (B_R _- B_F_) between the coefficient for plan from reduced equations omitting each mediator variable (B_R_) and the coefficient from the full model (B_F_).

Thus, results from analyses differentiating the direct effects of plan on overall satisfaction from indirect effects through compensation, claims service, and the quality of communication revealed that the configuration of factors accounting for overall differences in satisfaction were plan specific. Although compensation tended to play a central role in accounting for differences in overall satisfaction among plans, claims service played a major role in accounting for overall satisfaction with 2 of the plans.

## Discussion

This article is to our knowledge the first to have analyzed critical dimensions of dental practice satisfaction with PPOs and provides important new data on how dental managed care programs can maintain and improve participation in their plans. In contrast to findings among physicians [11], dental practices participating in dental managed care programs were relatively satisfied with the plans they rated, with average scores across plans falling between "very satisfied" and "satisfied." However, variability in overall satisfaction among plans was substantial, with 4 of the 6 plans assessed in this study differing significantly from the sample mean. Based on previous surveys of both physicians and dentists it is not surprising that compensation issues loomed large in explaining differences among perceptions of the plans, and for 4 of the 6 plans the perceived adequacy of compensation accounted for the lion's share of their differences in overall satisfaction. Yet compensation alone did not account for variability in overall satisfaction, as 2 of the plans' satisfaction ratings – one highly rated and the other the lowest rated – were substantially influenced by ratings of claims service. Finally, although a significant predictor of overall satisfaction, the quality of communication appeared to have a negligible role in explaining differences among plans in overall satisfaction.

In addition, results from this study indicate that practice patterns among dentists and participation in managed care may have changed substantially since the 1995 ADA survey of dentists' perceptions of dental managed care programs. In contrast with the ADA survey, data from the current survey indicate that dentists were heavily involved with dental managed care programs, with participants reporting being enrolled on average in almost 8 managed care plans. Furthermore, in 1995 dentists reported that joining a dental managed care plan was generally better for new dentists in order to increase their practice and was more common among younger dentists. Our study did not observe these trends, as participating practices were generally long-time, large, established enterprises. Participating practices also seemed to have generally more positive views of dental managed care with relatively good satisfaction ratings over all as well as favorable ratings of compensation. Dentists in the 1995 survey were highly dissatisfied with reimbursement levels and believed that managed care limited treatment options for patients. Although the divergent perceptions of managed care organizations in these two surveys may be attributable to different reporting sources – i.e., providers vs. office managers – these data do suggest that practice patterns with respect to managed care have changed markedly in the past decade.

Numerous studies of the impact of managed care on physicians' practices have revealed increasing levels of dissatisfaction with these insurance programs, particularly with respect to reimbursement and limits on professional autonomy. Although this same level of dissatisfaction was not observed in this study, it is possible that dental provider satisfaction will decline if the more heavily "managed" of managed care plans (e.g., dental health maintenance organizations [DHMOs]) achieve comparable levels of penetration in the dental insurance market as they have with medical insurance. Future studies should seek to identify those factors aside from compensation, service and communication that contribute to overall satisfaction with PPOs, and should also address questions that may be of greater salience to providers, as opposed to office managers. Issues concerning professional autonomy, limitations in clinical management of the patient, and utilization review could be crucial elements of provider satisfaction and might reveal trends among dentists that parallel those observed in the more extensive literature on physicians and managed care. An additional challenge relates to the need to further develop and refine measures of compensation, claims service, and plan communication. Substantial correlations (r = 0.48 – 0.66) among the three components of satisfaction analyzed in this study were observed; while this is likely attributable to the substantial conceptual overlap among these factors – i.e., does frustration with delayed payments manifest itself in compensation ratings or in ratings of claims service? – some portion of this may be due to measurement imprecision that blurs the distinction between constructs.

### Limitations

The results of this study must be considered in the context of its limitations. Although the sample is quite large (n = 4,582), the use of quota sampling limits the generalizability of results. Secondly, respondents in this study were typically office managers and not dentists. While the views of the office manager may reflect the general perceptions of the dentist, these results can only be applied to dental practices and not providers. Future research should seek to replicate and extend these finds with systematic probability samples of providers.

## Conclusion

The results of this study demonstrate the importance of compensation to dental practice satisfaction with particular dental PPOs. However, these results also highlight the critical role of service-related factors in differentiating plans and suggest that there are important non-monetary dimensions of PPO performance that can be used to recruit and retain practices.

## Competing interests

The authors declare that they have no competing interests. Although the authors have provided consulting services to MetLife, the blinding of dental plans in this analysis preclude the possibility of financial benefit (or loss) to MetLife or any other health insurer due to the publication of this manuscript.

## Authors' contributions

RHA, SR, and JK conceived of the study; RHA and SR were responsible for writing the manuscript; EAS conducted statistical analysis and contributed to writing and revising the manuscript; JK reviewed and revised the manuscript. All authors read and approved the final manuscript.

## Pre-publication history

The pre-publication history for this paper can be accessed here:


